# Respiratory syncytial virus infection in infants in rural Nepal

**DOI:** 10.1016/j.jinf.2016.05.007

**Published:** 2016-08

**Authors:** Helen Y. Chu, Joanne Katz, James Tielsch, Subarna K. Khatry, Laxman Shrestha, Steven C. LeClerq, Amalia Magaret, Jane Kuypers, Mark Steinhoff, Janet A. Englund

**Affiliations:** a Department of Medicine, University of Washington, Seattle, WA, USA; b Department of International Health, Johns Hopkins University, Baltimore, MD, USA; c Department of Global Health, George Washington University, Washington, D.C., USA; d Nepal Nutrition Intervention Project-Sarlahi, Sarlahi District, Nepal; e Department of Pediatrics and Child Health, Institute of Medicine, Tribhuvan University, Kathmandu, Nepal; f Department of Laboratory Medicine, University of Washington, Seattle, WA, USA; g Department of Global Health, Cincinnati Children's Hospital, Cincinnati, OH, USA; h Department of Pediatrics, University of Washington, Seattle Children's Research Institute, Seattle, WA, USA

**Keywords:** Respiratory syncytial virus, Pneumonia, Preterm birth, Resource-limited setting

## Abstract

**Objectives:**

Respiratory syncytial virus (RSV) pneumonia is a leading cause of infant mortality worldwide. The risk of RSV infection associated with preterm birth is not well-characterized in resource-limited settings. We aimed to obtain precise estimates of risk factors and disease burden of RSV in infants in rural southern Nepal.

**Methods:**

Pregnant women were enrolled, and along with their infants, followed to six months after birth with active weekly home-based surveillance for acute respiratory illness (ARI). Mid-nasal swabs were obtained and tested for RSV by PCR for all illness episodes. Birth outcomes were assessed at a postpartum home visit.

**Results:**

311 (9%) of 3509 infants had an RSV ARI. RSV ARI incidence decreased from 551/1000 person-years in infants born between 28 and 31 weeks to 195/1000 person-years in infants born full-term (p = 0.017). Of 220 infants (71%) evaluated in the health system, 41 (19%) visited a hospital or physician. Of 287 infants with an assessment performed, 203 (71%) had a lower respiratory tract infection.

**Conclusions:**

In a rural south Asian setting with intensive home-based surveillance, RSV caused a significant burden of respiratory illness. Preterm infants had the highest incidence of RSV ARI, and should be considered a priority group for RSV preventive interventions in resource-limited settings.

## Highlights

• RSV caused a significant burden of respiratory illness in infants in rural Nepal.
• The highest incidence of RSV was in preterm infants.
• Most infants with RSV had lower respiratory tract infection or a health care visit.
• Strategies are needed to prevent RSV in preterm infants in resource-limited settings.

## Introduction

Pneumonia is the primary infectious cause of death in children under age five worldwide. Respiratory syncytial virus (RSV) is the most common cause of viral pneumonia, and greater than 99% of deaths due to RSV are estimated to occur in resource-limited settings.^1^, ^2^ No vaccine is currently available for RSV, though several are under development for both infants and pregnant women. Precise estimates of baseline disease burden are needed to guide vaccine trials and set priorities for national immunization programs. Preterm birth, low birthweight, indoor air pollution, malnutrition, household crowding, and bacterial and parasitic infections have been shown to increase risk of severe pneumonia, and may influence morbidity and mortality due to RSV in resource-limited settings.^1^

Studies describing RSV in resource-limited settings have primarily used samples collected in clinics or hospitals. A large proportion of deaths due to respiratory disease or pneumonia may occur outside of these settings. This may lead to an underestimate of true population-based disease burden.^3^, ^4^ Additionally, many studies have not captured the impact of birth outcomes on RSV infection risk.^5^ Preterm birth is the primary risk factor for severe RSV disease in developed countries and is an indication for prophylaxis with palivizumab, a monoclonal antibody against RSV.^6^ However, the majority of preterm birth is estimated to occur in Africa and Asia. There is a lack of data from South Asia evaluating the association of preterm birth with RSV disease.

The objective of this study was to characterize the impact of prematurity and low birthweight on RSV acute respiratory illness (ARI) in very young infants from birth to six months of age using active weekly home-based surveillance for respiratory viral infections in Nepal.

## Materials and methods

Two consecutive, population-based, randomized, placebo-controlled trials of maternal influenza immunization were conducted in annual cohorts in the sub-tropical plains of rural southern Nepal from April 2011 to May 2014.^7^ Women were enrolled in the second to third trimester of pregnancy and followed, along with their infants, with weekly home-based visits until 180 days after birth. Clinical and sociodemographic data were collected at enrollment and at weekly visits. Birthweight was measured at a postpartum home visit. Infant ARI was defined as having any of the following: fever, cough, wheeze, difficult or rapid breathing, or a draining ear. For ARI episodes, health care worker collected mid-nasal swabs and performed an assessment of illness severity. Households were visited on a weekly basis, and swabs were collected if the infant met criteria for a respiratory illness in the past seven days. Swabs were transported in a temperature-stable nucleic acid buffer (Primestore, Austin, TX, U.S.A.) to the field laboratory, where they were aliquoted and stored at −80 °C until transport to the University of Washington Virology Laboratory in Seattle, WA, U.S.A. Virologic testing was performed for RSV using real-time polymerase chain reaction (qPCR).^8^

Data were analyzed using SAS 9.4 (Cary, NC). Incidence of RSV ARI was calculated using days of follow-up from birth until end of study or loss to follow-up. Days with symptoms as well as seven subsequent days were excluded from days at risk. An RSV epidemic period was defined as including weeks in which at least one case was detected, and at least three cases were found in any contiguous three-week period.^9^ Incidence rates were reported for RSV ARIs per 1000 person-years of observation overall and during RSV epidemic periods.

Birthweight was included if obtained <72 h after birth. Low birthweight was defined as <2500 g. Preterm birth was defined as <37 weeks completed gestation. Gestational age was calculated according to last menstrual period based on a house-to-house census conducted every five weeks of all women of childbearing age.^10^ If they had a missed period, then a urine pregnancy test was performed, and they were enrolled in the study if their urine pregnancy test was positive. Last menstrual period, therefore, was used to date the study with a five week recall period. Small-for-gestational-age (SGA) was defined according to the Alexander criteria.^11^ Z-scores were calculated using the WHO child growth standards.^12^ A household was defined as the persons sharing a common cookstove. Household density was the numbers of persons/room in the household. An RSV ARI was defined as the presence of any symptoms occurring for a minimum of one day with a mid-nasal swab obtained and RSV detected on qPCR assay. Illness episodes were considered unique if they were separated by seven symptom-free days. Only the primary RSV ARI was included in this analysis. Lower respiratory tract infection (LRTI) was defined according to the World Health Organization Integrated Management of Childhood Illness criteria as cough and/or difficulty breathing in association with age-specific tachypnea or wheezing. In addition to the above definition, LRTI also included severe or very severe pneumonia. Severe pneumonia was defined as cough and/or difficulty breathing with chest wall indrawing. Very severe pneumonia was defined as cough and/or difficulty breathing with lethargy or unconsciousness, convulsions, cyanosis or inability to feed/drink or vomiting everything. Upper respiratory tract infection (URTI) was defined as a RSV ARI that did not meet criteria for LRTI.^13^

Examination of risk factors for RSV ARI was performed using Poisson regression, using log time at risk as the offset. Because of the highly seasonal nature of RSV in our study, we performed a sensitivity analysis restricted to infants under surveillance during the RSV epidemic period. A multivariate Poisson regression model was performed to evaluate risk factors for RSV ARI, using backward elimination to result in a final, parsimonious model. Characteristics of infections occurring within two months versus two to six months of birth were compared using Wilcoxon tests for continuous measures and χ^2^ tests for categorical ones. Measures potentially associated with LRTI versus upper respiratory tract infections (URTI) were evaluated similarly. Poisson for correlated data, or a generalized estimating equation, was used to evaluate whether RSV ARI risk changed with age, by including each infant six times in the model, once for each month of life.

IRB approval for the study was obtained from the Johns Hopkins University Bloomberg School of Public Health, Seattle Children's Hospital, Cincinnati Children's Hospital, the Institute of Medicine at Tribhuvan University, and the Nepal Health Research Council. The trial in which this sub-study was conducted is registered at Clinicaltrials.gov (NCT01034254).

## Results

**Figure 1:**
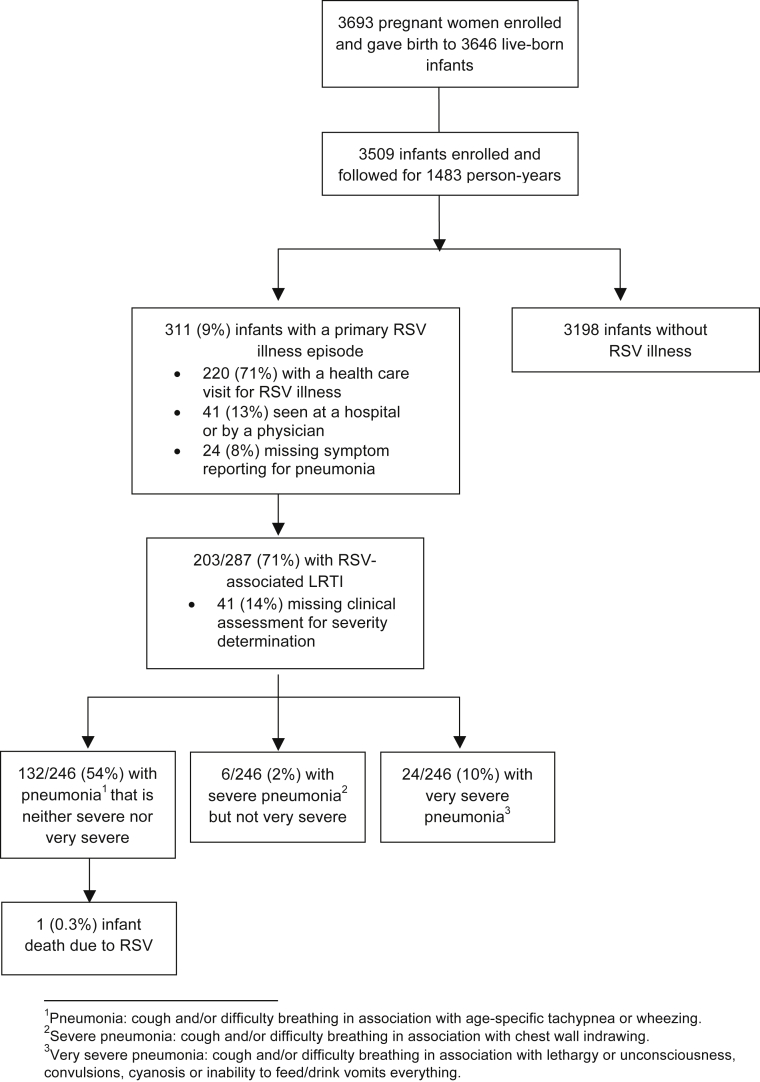
Flow diagram showing infants followed in the study from birth until 180 days of age.

Altogether, 3693 women enrolled during pregnancy and gave birth to 3646 live-born infants. A total of 3509 infants with weekly illness surveillance were followed for 1461 person-years. Over 75,000 weekly visits were conducted with collection of 4204 nasal swabs for infant ARI (Fig. 1). For all infants in the study, the median duration of surveillance was 180 days (IQR 170–180), with 0.5% of the children in the study having follow-up for less than 150 days. Approximately 90% of ARIs overall identified in infants had a swab taken, including 91% of those without fever and 89% of those with fever. In this cohort, 311 (9%) infants had a primary RSV ARI (Table 1).

**Figure 2:**
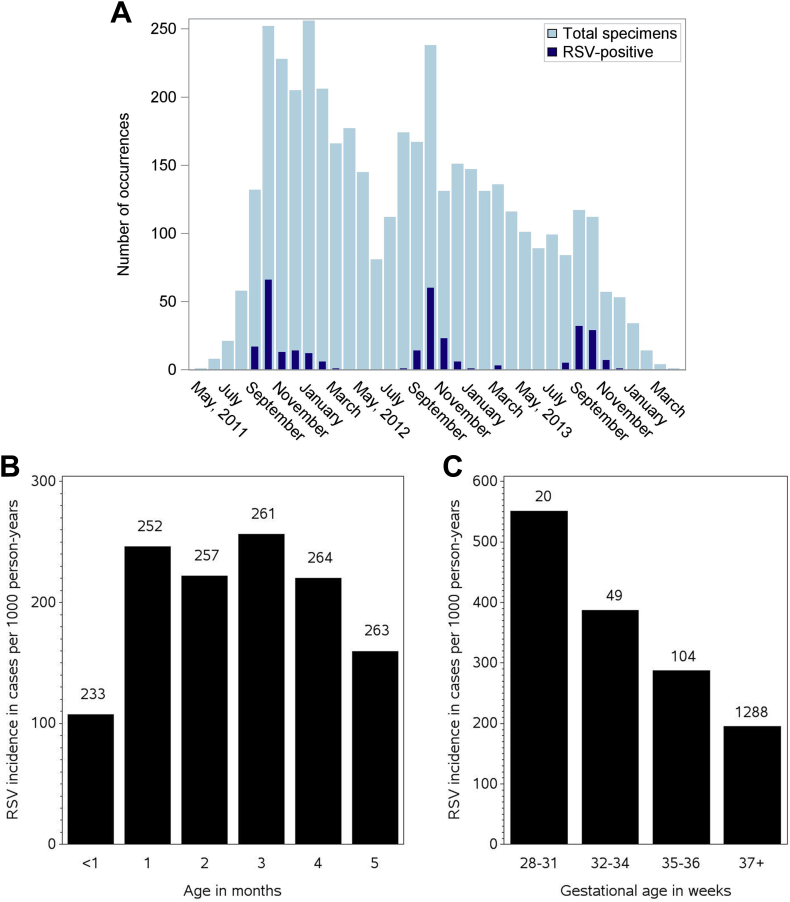
A. Seasonality of RSV over three seasons, from 2011 to 2014, in rural southern Nepal using active-home based surveillance. Overall incidence was 213/1000 person-years, while the incidence during RSV epidemic periods was 443/1000 person-years (by year, 2011–2012: 439/1000; 2012–2013: 407/1000; 2013–2014: 518/1000). B. Incidence based graph of RSV by age in months. The incidence of RSV ARI by age was lowest in the first month of life (107/1000 person-years) and higher in each of months two through five (246, 222, 256, and 220/1000 person-years, p < 0.001 for each), but not higher in month six (160/1000 person-years, p = 0.12). C. Incidence based graph of RSV by gestational age (very preterm: 28–31, preterm: 32–34, late-preterm: 35–36, term: >37). By gestational age, the incidence of RSV decreased from 551/1000 person-years in infants born between 28 and 31 weeks to 195/1000 person-years in infants born at 37 weeks or later (Fig. 2C, p = 0.017).

RSV was highly seasonal, with a peak between September and November over three years (Fig. 2A). Overall incidence was 213/1000 person-years, while the incidence during RSV epidemic periods was 443/1000 person-years (by year, 2011–2012: 439/1000; 2012–2013: 407/1000; 2013–2014: 518/1000). The incidence of RSV ARI by age was lowest in the first month of life (107/1000 person-years) and higher in each of months two through five (246, 222, 256, and 220/1000 person-years, p < 0.001 for each), but not higher in month six (160/1000 person-years, p = 0.12; Fig. 2B). By gestational age, the incidence of RSV decreased from 551/1000 person-years in infants born between 28 and 31 weeks to 195/1000 person-years in infants born at 37 weeks or later (Fig. 2C, p = 0.017). Thirty (10%) infants with RSV were born at a gestational age of 34 weeks or less.

On bivariate analysis, the single maternal factor associated with risk of RSV ARI was maternal education (Incidence rate ratio (RR): 0.95; 95% CI: 0.91–0.99; Table 1). Household factors included use of an open indoor biomass cookstove (IRR: 2.00; 95% CI: 1.10–3.64), and presence of a household latrine (IRR: 0.69; 95% CI: 0.47–1.00). Other factors not significantly associated were number of persons per room (IRR: 1.01; 95% CI: 0.96–1.06), number of other children (IRR: 1.09; 95% CI: 0.98–1.20), and number of other children under age five (IRR: 1.22; 95% CI: 0.99–1.51). Infant factors included preterm birth (IRR: 1.78; 95% CI: 1.12–2.83), and birth during monsoon season (typically late June to end of September; IRR: 5.34; 95% CI: 3.36–8.47). The risk of RSV ARI was not different between preterm infants who were or were not SGA (p = 0.39).

We conducted a sensitivity analysis restricted to the RSV epidemic period in which 47% of follow-up (688/1461 person-years) and 98% of infections (305/311) occurred. Bivariate results are strikingly similar, though the increase in RSV ARI associated with monsoon season correspondingly decreased from an IRR of 5.34 to 2.60 (Table 1). On multivariate analysis of the full risk period initially including maternal education, maternal literacy, use of open cookstove, children under age five, household latrine, gestational age and monsoon season, the only risk factors that remained significant for RSV ARI were maternal years of education (IRR: 0.95; 95% CI: 0.91–0.99) and monsoon season of birth (IRR: 5.34; 95% CI: 3.32–8.58).

The median age at RSV ARI was 12 weeks (IQR, 7–17 weeks). As compared to older infants, infants with a primary RSV ARI under age two months had similar duration of fever, wheezing or difficulty breathing (Table 2). A slight difference was seen between infants < two months versus two to six months in days of cough (three vs. four, respectively; p = 0.035). Most infants with RSV ARI had a health care visit for their illness (n = 220; 71%). Of these, 41 (13%) infants were seen at a hospital (n = 4) or by a physician (n = 37), while the rest were seen at a local health facility, by a local healer or at a medicine shop. Overall infant hospitalization rate was 2.7/1000 person-years. One (1%) infant died following RSV ARI, while 46 (1%) of infants without RSV ARI died of other etiologies, including pneumonia, diarrhea, preterm birth and congenital abnormalities. The one death was a 7 week old male infant born at 31 weeks gestation. He had five days of fever, cough, and difficulty breathing, and was seen for care at a health care facility and prescribed antibiotics. His caregiver reported that after being put to bed, he was found to have stopped breathing one hour later.

**Figure 3:**
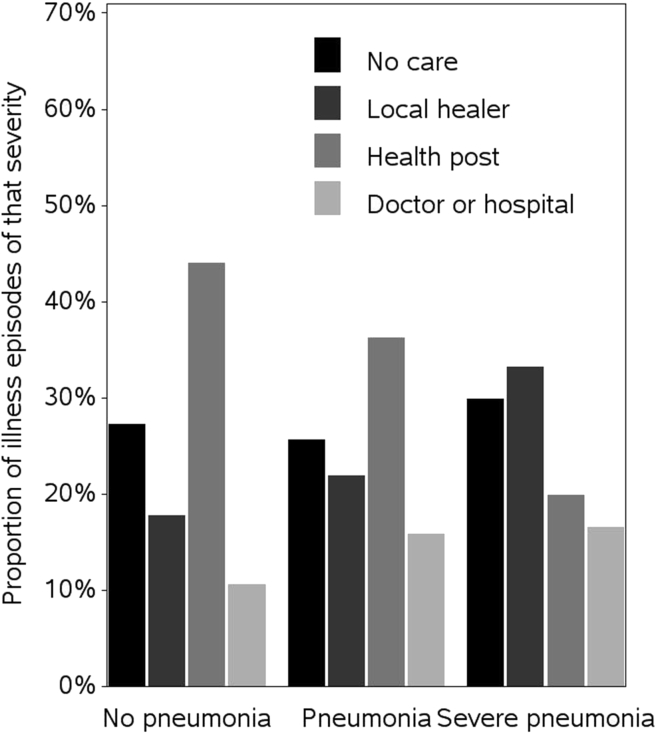
Relationship between disease severity and health-care seeking among infants with RSV infection. Infants with LRTI were no more likely to seek a higher level of care for their illness than those with an URTI (p value for trend = 0.85).

Of 311 infants with RSV ARI, 287 (92%) had family-reported symptoms by which to assess LRTI, and 246 (79%) had a clinical assessment performed by study workers trained to collect signs and symptoms of respiratory illness. A total of 203 of 287 (71%) of infants had LRTI by any criterion. Of the 246 infants with a clinical assessment, six (2%) had severe pneumonia and 24 (10%) had very severe pneumonia. Those with very severe pneumonia had the following qualifying criteria: inability to feed/drink (n = 21; 88%), chest indrawing (n = 7; 29%), or lethargy/unconsciousness (n = 5; 21%). Infants with LRTI were no more likely to seek a higher level of care for their illness than those with an URTI (p value for trend = 0.85; Fig. 3).​ When comparing infants with and without LRTI, no significant differences were seen in maternal education, number of children in the household, or use of an open indoor cookstove (Table 3). A trend was seen towards increased numbers of preterm infants with LRTI.

Of the 2622 infants with growth measured at six months, no significant difference in six month weight or length was observed between surviving infants with and without RSV ARI or when comparing infants with RSV-associated URTI vs. LRTI (Table 1, Table 3). The Z-scores of infants with RSV averaged −1.1 for weight-for-age, as compared to −1.0 for weight-for-age in those without RSV ARI (p = 0.63).

## Conclusions

In this large prospective population-based study using active home-based weekly surveillance of infants in rural Nepal, 9% of infants experienced a symptomatic RSV acute respiratory illness in the first six months of life. Most infants with RSV had a lower respiratory tract infection or accessed the health care system. The incidence of RSV ARI was highest in preterm infants, and decreased significantly with increasing gestational age. Our results quantify a significant burden of disease that could be mitigated through direct interventions, such as maternal vaccination.

In this study, mothers were surveyed for pregnancy every five weeks, allowing for accurate dating of gestational age in a region of the world where prenatal care, including first trimester ultrasound, is not routinely available. In addition, the majority of birthweights were measured <72 h after birth in a setting where ∼60% of mothers deliver at home. These aspects of the study design allow for an accurate assessment of risk for RSV ARI in preterm, low birthweight infants in rural south Asia. We find that the incidence of RSV ARI was highest in preterm infants, and decreased with increasing gestational age. Moreover, 10% of the cases of RSV ARI occurred in infants born at 34 weeks gestation or younger. Similar findings been described in hospital-based studies conducted in developed countries but not previously well described in a community-based, rural setting.^14^

Our results suggest preterm infants should be a priority group for RSV preventive interventions in resource-limited settings. Importantly, as decisions are made about optimal timing of maternal RSV vaccine administration, it will be important to evaluate the role of preterm birth on RSV antibody transfer and protection from severe lower respiratory tract disease in specific populations. Preterm infants have less transplacental transfer of maternal antibody and more immature lungs compared to full-term infants, making them more susceptible to severe lower respiratory tract illness.^15^ A strategy to vaccinate women earlier in pregnancy may better protect the group with the highest incidence of RSV ARI.

Longitudinal household-based weekly surveillance was conducted over three full calendar years to accurately estimate the incidence of symptomatic RSV ARI in infants from birth to six months of age. The use of active surveillance on a weekly basis minimizes recall bias, particularly in households where multiple young children are present.^16^ In this study, 9% of infants had at least one RSV ARI episode, with an incidence of 213/1000 person-years overall and 443/1000 person-years during RSV epidemic periods. This compares to incidence rates of 70/1000 person-years in children between 6 months and 10 years of age in Australia, Singapore, and Colombia,^17^ 345/1000 person-years in Kenyan infants,^18^ and 300/1000 person-years in rural Peru.^19^ RSV was highly seasonal with clear epidemics over three years. Because of this marked seasonality, season of birth was the primary risk factor for acquiring RSV. This degree of seasonality is not seen in tropical regions for influenza, and has not been found to be the case in other tropical regions, such as Taiwan and Singapore, for RSV.^20^ This demonstrates the need for year-round respiratory viral surveillance data in the specific region of interest to guide public health strategies.

Risk of RSV ARI was impacted by factors common in poor rural communities, including lack of a household latrine, use of open indoor cookstoves and multiple young children in the household. For infants born during the RSV epidemic period, maternal education was associated with protection. Maternal education is likely a proxy for general socioeconomic status and health care access, and not causally associated with RSV ARI risk. There was no association found between years of education and care-seeking among mothers of kids with RSV. Though we did not find an association of RSV infection with open biomass cookstove use, only a minority of households used non-biomass cookstoves limiting the power to detect an association. Biomass stoves have been linked to increased risk for pneumonia in children; however in a clinical trial in Guatemala replacement of open cookstoves reduced risk of severe pneumonia but did not reduce cases of RSV pneumonia.^21^ We also did not find that other young children in the same household increased RSV ARI risk; this may be due to the high degree of intermixing of children between households in dense, crowded village settings. In U.S.-based epidemiologic studies, older siblings likely introduce RSV to the infant.^22^, ^23^ Likewise in Kenya, molecular epidemiology studies have linked identical strains of RSV in older children to the infants in the same household. In our setting, vaccination of older children may not be a sufficient strategy to protect young infants in the same household.

A consideration in the performance of RSV vaccine trials is the selection of clinical endpoints, including the use of medically attended illness as an indicator of disease severity.^24^, ^25^ In resource-limited settings, health care access is impacted by several factors unrelated to illness severity, including maternal education, poverty, distance to a health care facility, cost related to transportation and care, and gender.^26^ The use of medically attended illness as a clinical endpoint would potentially exclude participation of large sectors of the population and thereby delay the introduction of an effective intervention. Though we found a trend for seeking care at a hospital or from a physician in infants with RSV LRTI, several infants with severe pneumonia did not seek any care. A combination of a clinical evaluation with health-care seeking behavior would be more likely to accurately stratify disease severity.

We additionally compared the illness characteristics of infants with RSV ARI between birth and two months to those with a primary illness episode between two and six months of age. Infants under two months of age are potentially protected by higher levels of maternal antibody. However, we did not find that younger infants had increased rates or duration of symptoms compared to older infants. It is possible that other factors, like malnutrition and hypergammaglobulinemia, may lessen the transfer of RSV-specific antibody and mitigate its early protective effect, as in mother–infant pairs in Papua New Guinea.^27^

Limitations of this study included the lack of collection of swabs in asymptomatic infants, leading to an underestimation of the true incidence of RSV ARI. However, unlike other respiratory viruses such as rhinovirus, a primary RSV illness episode in a very young infant is rarely asymptomatic.^28^ Additional limitations of the study included the lack of rhinorrhea as part of the definition of acute respiratory illness. This likely reduced the detection of acute symptomatic RSV infection in young infants, in particular in those without lower respiratory tract infections. Sampling was performed only once a week, so it is also possible that RSV was not detected in certain illness episodes with viral shedding that lasted less than one week. One potential explanation why premature infants had a higher incidence of RSV infection is that they are more likely to have severe infection, and therefore shed virus for prolonged periods of time, as has been shown with immunocompromised adults. This would explain why detection would be more likely in this population since sampling was performed only on a weekly basis. An additional limitation was the lack of pulse oximetry or chest radiographs to evaluate for pneumonia. Severe or very severe pneumonia was defined according to the WHO IMCI criteria; we believe the use of this definition is likely to be comparable across multiple studies in resource-limited settings. Finally, the lack of first trimester ultrasound was not available for accurate dating of gestational ages; this was not possible in rural Nepal and in parts of the world where the majority of women receive limited antenatal care and the majority of births occur at home.

Prevention of RSV in resource-limited settings has the potential to significantly decrease severe illness in infants under six months of age, particularly among those born preterm. Further studies are needed to evaluate the optimal strategy to prevent RSV in high-risk preterm infants in resource-limited settings.

## Contributors

HYC designed the study with major inputs from JAE, JKa, MS, and JT. JT, JKa, MS, SK, SC, and LS were involved in study design, recruitment, and data collection in Nepal. JKu performed the laboratory testing. HYC and AM performed the descriptive and univariate analyses, AM did the multivariate analyses and checked all of the analyses. HYC wrote the first draft, with major contributions from JAE, and all authors reviewed subsequent drafts and agreed on the final version of the paper.

## Conflict of interests

JAE has received research support from Gilead, Chimerix, GlaxoSmithKline, Pfizer, and Roche, and serves as a consultant for GlaxoSmithKline, Pfizer, and Gilead. HYC has received research support from GlaxoSmithKline. MS serves on the board of the Novartis Vaccine Institute for Global Health. All other authors declare no conflicts of interest. This work was supported by National Institutes of Health grants K23-AI103105 (HYC), PATH (HYC, JAE), and Bill and Melinda Gates Foundation Grant 50274 (all authors).

## Figures and Tables

**Table 1:** Risk factors for infant RSV acute respiratory illness overall and during RSV season.

Risk factors for RSV	Characteristic n (%) or median (IQR)		Overall follow-up		RSV season only	
	Uninfected (n = 3198)	RSV-infected (n = 311)	IRR (95% CI)	p-Value	IRR (95% CI)	p-Value
Maternal primiparity	1355 (42.5%)	109 (35%)	0.75 (0.51, 1.10)	0.14	0.78 (0.54, 1.12)	0.18
Maternal age (n = 3301)	23 (20–26)	22 (20–26)	1.00 (0.96, 1.04)	0.99	0.99 (0.96, 1.04)	0.79
Maternal education (yrs) (n = 3237)	5 (0–10)	0 (0–8)	0.95 (0.91, 0.99)	0.020	0.95 (0.91, 0.99)	0.018
Maternal smoking (n = 3238)	93/2960 (3.1%)	12/278 (4.3%)	1.36 (0.53, 3.49)	0.52	1.41 (0.57, 3.50)	0.46
Maternal literacy (n = 3239)	1819/2961 (61.4%)	148/278 (53.2%)	0.72 (0.49, 1.06)	0.092	0.71 (0.49, 1.04)	0.078
Number of children under age 15 in household	2 (1–3)	2 (1–3)	1.09 (0.98, 1.20)	0.10	1.07 (0.97, 1.18)	0.15
Number of children under age five in household	0 (0–1)	1 (0–1)	1.22 (0.99, 1.51)	0.064	1.19 (0.96, 1.46)	0.11
Madeshi ethnicity	1302 (42.3%)	135 (45.5%)	1.18 (0.81, 1.71)	0.39	1.16 (0.80, 1.66)	0.43
Number of persons in household	7 (5–10)	7 (5–10)	1.01 (0.96, 1.06)	0.62	1.01 (0.96, 1.05)	0.77
Open indoor cookstove	2474 (80.3%)	266 (89.3%)	2.00 (1.10, 3.64)	0.023	1.88 (1.03, 3.45)	0.041
Number of persons/room	2.75 (2–4)	3 (2–5)	1.05 (0.98, 1.12)	0.15	1.04 (0.97, 1.10)	0.26
Household latrine	1554 (50.5%)	121 (40.7%)	0.69 (0.47, 1.00)	0.053	0.70 (0.48, 1.02)	0.061
Brahmin caste	341 (11.1%)	26 (8.8%)	0.78 (0.40, 1.51)	0.46	0.73 (0.39, 1.40)	0.35
Male sex of infant	1676 (52.4%)	175 (56.3%)	1.16 (0.80, 1.68)	0.44	1.13 (0.80, 1.61)	0.49
Gestational age, weeks	39.7 (38.4–40.9)	39.4 (37.9–40.7)	0.92 (0.85, 0.98)	0.017	0.92 (0.86, 0.99)	0.027
Preterm birth	375 (11.7%)	60 (19.3%)	1.78 (1.12, 2.83)	0.014	1.70 (1.09, 2.66)	0.019
Birthweight, kg (n = 2738)	2.8 (2.5–3.08)	2.76 (2.45–3.07)	0.78 (0.51, 1.21)	0.27	0.88 (0.58, 1.33)	0.54
Low birthweight (n = 2738)	606/2479 (24.4%)	72/259 (27.8%)	1.21 (0.78, 1.89)	0.39	1.14 (0.75, 1.73)	0.54
Monsoon season birth (birth between June–Sept)	1299 (40.6%)	248 (79.7%)	5.34 (3.36, 8.47)	<0.0001	2.60 (1.66, 4.08)	<0.0001
Small for gestational age (n = 2737)	1354/2478 (54.6%)	140/259 (54.1%)	0.99 (0.67, 1.48)	0.97	0.92 (0.63, 1.34)	0.67
Breastfeeding at birth	2624 (82.3%)	260 (83.6%)	0.97 (0.59, 1.58)	0.89	0.93 (0.58, 1.48)	0.76
Six month weight, kg (n = 2622)	6.8 (6.2–7.4)	6.7 (6.2–7.4)	0.96 (0.79, 1.18)	0.73	0.97 (0.80, 1.18)	0.79
Six month length, cm (n = 2621)	64.4 (62.7–66.1)	64.3 (62.1–65.8)	0.96 (0.90, 1.03)	0.29	0.96 (0.90, 1.02)	0.21
6 mo Z score for weight (n = 2622)	−1.0 (−1.8 to −0.3)	−1.1 (−1.9 to −0.4)	0.95 (0.80, 1.12)	0.53	0.96 (0.82, 1.13)	0.63
6 mo Z score for length (n = 2621)	−1.0 (−1.8 to −0.3)	−1.2 (−1.9 to −0.5)	0.90 (0.78, 1.05)	0.17	0.89 (0.77, 1.03)	0.13
Mortality	46 (1.4%)	1 (0.3%)	0.91 (0.04, 23.21)	0.96	0.79 (0.04, 16.79)	0.88

* Ns and denominators shown when at least 5% of data missing overall for that characteristic.

**Table 2:** RSV illness episode characteristics in infants with RSV acute respiratory illness from birth to two months compared to those age 2–6 months.

RSV illness episode characteristics Median (min, max) or n (%)	Birth to 2 months (n = 106)	2–6 months (n = 205)	p-Value
Days of symptoms			
Fever	1 (0–11)	2 (0–16)	0.10
Cough	3 (0–17)	4 (0–19)	0.035
Wheeze	2 (0–25)	3 (0–11)	0.66
Difficulty breathing	2 (0–13)	2 (0–12)	0.60
Any symptoms	4 (0–26)	5 (0–21)	0.15
Visit for care (Y/N)	31 (29)	60 (29)	0.29^a^
No visit			
Medicine shop or local doctor	17 (16)	51 (25)	
Sub-health post or health post	41 (39)	70 (34)	
Doctor, nursing home or hospital	17 (16)	24 (12)	
WHO Integrated Management of Childhood Illness criteria	n = 82	n = 164	
Chest indrawing	8 (10)	6 (4)	0.052
Lethargy/unconsciousness	3 (4)	2 (1)	0.21
Convulsions	0 (0)	0 (0)	–
Cyanosis	1 (1)	1 (1)	0.62
Inability to feed/drink or vomits everything	10 (12)	11 (7)	0.16
Lower respiratory tract infection (n = 203)	70 (71)	133 (71)	0.99
Severe or very severe pneumonia (n = 30)	14 (17)	16 (10)	0.098
Very severe pneumonia (n = 24)	11 (13)	13 (8)	0.17
6 mo Z-score for weight (median (IQR))	−1.4 (−2.0, −0.6)	−1.1 (−1.9, −0.4)	0.33
6 mo Z-score for length (median (IQR))	−1.2 (−1.7, −0.8)	−1.3 (−1.9, −0.3)	0.98

a χ^2^ test for trend.

**Table 3:** Factors associated with lower respiratory tract infection in infants with RSV.

Characteristics Median (IQR) or n (%)	Upper respiratory tract infection (n = 84)	Lower respiratory tract infection (n = 203)	p-Value
Male gender	50 (60)	110 (54)	0.41
Low birthweight	25 (33)	47 (29)	0.45
Preterm birth	11 (13)	47 (23)	0.055
Small for gestational age	45 (60)	87 (53)	0.30
Other children under age five in household	44 (54)	104 (55)	0.90
Open indoor cookstove	72 (88)	176 (92)	0.32
Household latrine	34 (42)	70 (37)	0.45
Maternal primiparity	34 (41)	66 (32)	0.20
Maternal literacy	46 (60)	89 (50)	0.17
Visit for care			0.85^a^
No visit	23 (27)	58 (29)	
Medicine shop or local doctor	15 (18)	47 (23)	
Sub-health post or health post	37 (44)	66 (33)	
Six-month Z-score for weight	−1.1 (−2.0, −0.5)	−1.1 (−1.9, −0.2)	0.58
Six-month Z-score for height	−1.2 (−1.9, −0.8)	−1.3 (−1.9, −0.4)	0.59

a χ^2^ test for trend.
